# SARS-CoV-2: An Overview of the Genetic Profile and Vaccine Effectiveness of the Five Variants of Concern

**DOI:** 10.3390/pathogens11050516

**Published:** 2022-04-26

**Authors:** Raluca Dumache, Alexandra Enache, Ioana Macasoi, Cristina Adriana Dehelean, Victor Dumitrascu, Alexandra Mihailescu, Roxana Popescu, Daliborca Vlad, Cristian Sebastian Vlad, Camelia Muresan

**Affiliations:** 1 Ethics and Human Identification Research Center, Department of Neurosciences, “Victor Babes” University of Medicine and Pharmacy, 300041 Timisoara, Romania; raluca.dumache@umft.ro (R.D.); enache.alexandra@umft.ro (A.E.); alexandra.mihailescu@umft.ro (A.M.); muresan.camelia@umft.ro (C.M.); 2 Departament of Toxicology and Drug Industry, Faculty of Pharmacy, “Victor Babeş” University of Medicine and Pharmacy Timisoara, Eftimie Murgu Square No. 2, 300041 Timişoara, Romania; 3 Research Center for Pharmaco-Toxicological Evaluations, Faculty of Pharmacy, “Victor Babes” University of Medicine and Pharmacy Timisoara, Eftimie Murgu Square No. 2, 300041 Timisoara, Romania; 4 Department of Pharmacology and Biochemistry, Discipline of Pharmacology, “Victor Babes” University of Medicine and Pharmacy, 300041 Timisoara, Romania; dumitrascu.victor@umft.ro (V.D.); vlad.daliborca@umft.ro (D.V.); vlad.cristian@umft.ro (C.S.V.); 5 Genetics, Genomic Medicine Research Center, Department of Microscopic Morphology, “Victor Babes” University of Medicine and Pharmacy, 300041 Timisoara, Romania; 6 Department of Microscopic Morphology, Discipline of Molecular and Cell Biology, “Victor Babes” University of Medicine and Pharmacy, 300041 Timisoara, Romania; popescu.roxana@umft.ro

**Keywords:** SARS-CoV-2, evolution, mutagenesis, Spike protein, genetic variation

## Abstract

With the onset of the COVID-19 pandemic, enormous efforts have been made to understand the genus SARS-CoV-2. Due to the high rate of global transmission, mutations in the viral genome were inevitable. A full understanding of the viral genome and its possible changes represents one of the crucial aspects of pandemic management. Structural protein S plays an important role in the pathogenicity of SARS-CoV-2, mutations occurring at this level leading to viral forms with increased affinity for ACE2 receptors, higher transmissibility and infectivity, resistance to neutralizing antibodies and immune escape, increasing the risk of infection and disease severity. Thus, five variants of concern are currently being discussed, Alpha, Beta, Gamma, Delta and Omicron. In the present review, a comprehensive summary of the following critical aspects regarding SARS-CoV-2 has been made: (i) the genomic characteristics of SARS-CoV-2; (ii) the pathological mechanism of transmission, penetration into the cell and action on specific receptors; (iii) mutations in the SARS-CoV-2 genome; and (iv) possible implications of mutations in diagnosis, treatment, and vaccination.

## 1. Introduction

Humanity has been struggling with one of the most difficult pandemics, both in terms of prevention and treatment, since the end of 2019. The first case of coronavirus disease (COVID-19) was reported in December 2019 in Wuhan, China. Severe Acute Respiratory Syndrome (SARS) Coronavirus 2 (SARS-CoV-2) has been identified as the pathogen responsible for this respiratory illness, originally referred to by the World Health Organization as the 2019 novel coronavirus (2019-nCoV) [1]. 

SARS-CoV-2 belongs to the genus beta-coronavirus of the family Coronaviridae [2], being the ninth type of coronavirus documented as causing infections in humans [3]. Infections by Coronaviruses can occur in both humans and animals. Coronaviruses are heterogeneous, potentially fatal viruses. In the early 2000s, mankind was confronted with two pathogens of this class of viruses, namely, Severe Acute respiratory Syndrome Coronavirus (SARS-CoV) and Middle East Respiratory Syndrome Coronavirus (MERS-CoV), causing many severe infections and even death to humans [4].

Coronaviruses are enveloped viruses with the longest positive RNA genome of any RNA virus, causing infections in humans and other mammals and birds [5]. There is a high degree of similarity between the SARS-CoV-2 genome and other betacoronaviruses, accounting for 79% of the SARS-CoV genomic sequence and 50% of the MERS-CoV genomic sequence [6]. Although coronaviruses generally cause mild and moderate respiratory infections, some representatives of the class such as SARS, MERS and SARS-CoV-2 are associated with an increased incidence of lethality [5]. So far, there have been more than 447 million infections caused by SARS-CoV-2 and more than 6 million deaths caused by the virus until 2022.

**Figure 1:**
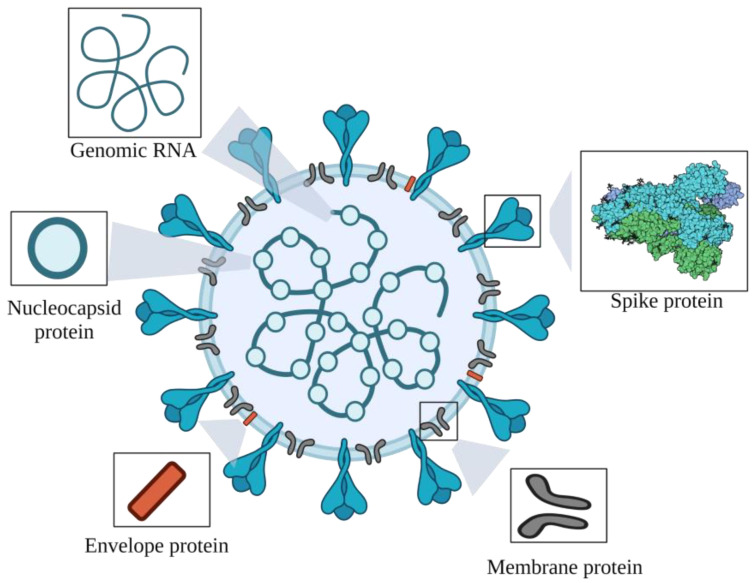
Graphical representation of the RNA genome and proteins of respiratory syndrome coronavirus 2 (SARS-CoV-2). Created with BioRender.com (accessed on 30 March 2022).

SARS-CoV-2 is an encapsulating virus that carries a single stranded RNA. In terms of structure, the virus resembles a corona, consisting of a double-layered lipid shell, the spike glycoprotein (S), the envelope protein (E), the membrane glycoprotein (M) and the nucleocapsid protein (N) (Figure 1) [7]. Spike glycoprotein is responsible for interacting with host cell receptors [8], while membrane glycoprotein is responsible for assembling viral particles [9], nucleocapsid protein is involved in viral genome replication and cell signaling [10] and envelope protein plays a key role in viral pathogenicity [11]. The SARS-CoV-2 virus targets two lung cell types: goblet cells and cilia cells. Goblet lung cells are responsible for the production of mucus, while cilia cells are primarily responsible for removing debris from the lungs, making them ideal targets for viruses [12]. 

Once inside the cell, the virus releases viral RNA that can replicate on its own due to interference with cellular replication mechanisms. In addition, they produce proteins needed for the formation of new viruses, proteins that will be transported through Golgi bodies. The process of apoptosis occurs when the cells no longer maintain cellular homeostasis [1]. Thus, dead cells accumulate in the airways leading to lung damage, pneumonia and eventually complete respiratory failure and death of the patient [13]. 

Throughout the pandemic, scientists have made great efforts to fully understand the viral genome, as well as to identify any variants that may occur [14]. Due to the fact that SARS-CoV-2 is an RNA virus, it naturally undergoes mutations that lead to the formation of new viral forms. Depending on the type and location of the mutation, different forms may develop with a higher or lower risk of infection. For example, certain mutations in the Spike protein can alter the virus to enter the cell and decrease the effectiveness of the antibodies [15]. Protein S is one of the best characterized proteins of the Coronaviridae family, the mutations occurring at this level being closely related to the rate of human-to-human transmission [16].

Mutations in RNA viruses can be neutral, beneficial or harmful, most of which are neutral. However, some mutations can have an impact on viral replication and infectivity. Currently, the attention of researchers in the field has been directed to the monitoring of genetic changes in the genus SARS-CoV-2 to understand the biological impact of these new viral forms [17]. This review provides an extensive synthesis of the mutations that have emerged over the last two years of the pandemic, as well as discusses the implications of these mutations in terms of symptoms, transmission, treatment and immune response.

## 2. The SARS-CoV-2 Genome

As a member of the β-coronavirus (β-CoV) family, SARS-CoV-2 exhibits the genetic characteristics of this class of viruses. Namely, the viral RNA genome has a 5′ cap structure and 3′ poly-A tail, which allows it to function as an mRNA for translating replicase polyproteins [18]. Following initial genomic screening, SARS-CoV-2 was found to have a gene sequence similar to SARS-CoV and MERS-CoV, being more similar to SARS-CoV. In addition, recent studies have shown that SARS-CoV-2 has over 96% homology to BatCoV RaTG13, a type of coronoavirus found in *Rhinolophus affinis* bats [19]. 

SARS-CoV-2 has a single-stranded, RNA-positive single-stranded genome of approximately 30,000 nucleotides. Nucleocapsid proteins surround and encase the genome in ribonucleotide complexes. Additionally, the complex is enclosed by a lipid membrane composed of structural proteins S, M, and E [2]. 

**Figure 2:**
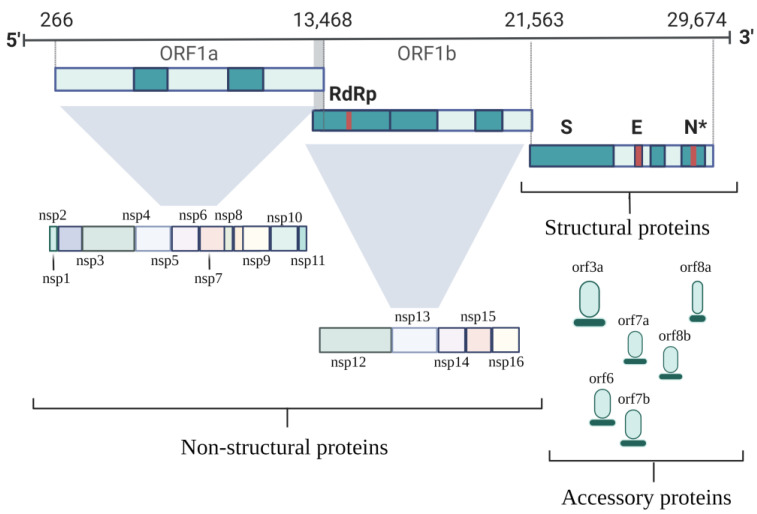
Organization of the SARS-CoV-2 genome. The genome has two large genes, ORF1a and ORF1b, which are involved in encoding 16 nonstructural proteins (nsp1-nsp16). Structural genes encode structural proteins: S, E and N proteins. In terms of accessory proteins (orphans) they are unique in number, genomic organization, sequence and function. Created with BioRender.com (accessed on 29 March 2022).

The genetic structure of SARS-CoV-2 consists of 12 functional open reading frames (ORFs), their arrangement being very similar to that found in SARS-CoV and MERS-CoV [20]. These genetic sequences have a variable length between 29.8 kb and 29.9 kb, at which the ORFs involved in the coding of 27 proteins are found [21]. Thus, the SARS-CoV-2 viral proteins, in addition to the S, M and E proteins, also comprise two large polyproteins (ORF1a and ORF1ab) and at least six accessory proteins (ORF3a, ORF6, ORF7a, ORF7b, ORF8a and ORF8b) (Figure 2) [15]. 

ORF1a and ORF1ab are involved in the coding of non-structural proteins (NSPs), called polyproteins 1a (pp1a) and polyproteins 1ab (pp1ab), respectively. Thus, polyprotein 1a comprises NSP1 to NSP 11, and polyprotein 1ab comprises NSP 12 to NSP16. NSPs’ activity has been documented, noting that they play an important role in the immunological suppression of host cells, being also essential for genome expression control and viral replication [22].

In the 3’ end of the genome, structural proteins S, E, M and N are encoded, while oppositely, at the 5’ end, there is a leader sequence and an untranslated region (UTR), which are responsible for stem loop structures, replication functions and transcription of the viral genome [23]. 

S, M and E proteins are incorporated into the viral membrane and are involved in the formation of virions. Protein S is a trimeric protein that has the ability to bind specifically to the cellular receptor, the enzyme converting angiotensin 2 (ACE2), promoting the virus’s entry into cells [24]. Protein E is involved in pathogenicity by forming an ion channel in the viral membrane [25], while protein N binds and packs viral genomic RNA as a ribonucleoprotein complex into virions, and protein M interacts with proteins S, E and N being involved in viral morphogenesis [26]. 

## 3. The Pathological Mechanism of SARS-CoV-2

### 3.1. Transmission

In order to stop the spread of SARS-CoV-2 and, ultimately, to stop the pandemic, it is crucial to understand the way the disease is transmitted. Currently, the literature suggests that the transmission of the virus is predominantly through respiratory drops. The transmission process can be affected by various environmental factors, such as temperature, humidity or air currents. The main routes of transmission of SARS-CoV-2 among the population will be discussed below [27].

*Respiratory Transmission.* The main route of transmission of SARS-CoV-2 is respiratory, which can be achieved mainly through respiratory drops, but also through aerosols that are released during coughing and sneezing [28]. In terms of aerosol transmission, an important feature is their size. Drops with a size less than 5 μM can be transmitted much more easily and remain viable for a longer period of time compared to larger particles [29]. The study by van Doremalen et al. suggested that SARS-CoV-2 has a viability of approximately 3 h in aerosols smaller than 5 μM. The study, however, exhibited two major limitations, namely, that the generation of aerosols was done mechanically, and the viral load was one of 50% tissue Culture Infectious Dose (TCID50) of 10^5.25^ per milliliter. Therefore, it is not clear whether these data are comparable to those of patients with COVID-19 [30]. The 50% tissue Culture Infectious Dose (TCID50) of companionship and air flow are the major factors contributing to respiratory viral transmission. Shen et al. investigated a group of 31 people who participated in an open-air religious ceremony, 24 of whom traveled by bus. Specifically, the researcher pointed out that the group that traveled by bus tested positive for the infection in 35% of the cases, as none of the other seven participants showed signs of infection. Thus, the major role played by poor ventilation in transmitting the infection was emphasized [31]. A similar study investigated contacts between train passengers, with more than 2000 cases of infection and more than 72,000 contacts, showing that the incidence of cases is closely related to the distance between places and the duration of the journey [32]. Another study found that the frequency of cases in gyms is closely related to the distance between athletes, the incidence being higher in individuals who undertake intense physical activities in a crowded room compared to individuals who take Pilates classes with a presymptomatic instructor in a less crowded environment [33]. Based on these studies, classical airway transmission appears to be more important than aerosol transmission. In addition, studies have found that wearing a mask indoors significantly reduces the risk of transmission, emphasizing once again the dominant role of the respiratory spread of this virus [34]. 

*Direct Contact.* It has been emphasized from the start of the pandemic that direct contact with the fomites that contain SARS-CoV-2, as well as subsequent contact with the biological mucous membrane, plays an important role in transmitting this disease [27]. In several studies, the SARS-CoV-2 virus has been evaluated for its survival rates on various surfaces. These studies concluded that the virus lives between four and three days on copper and plastic, respectively [35]. Ong and its collaborators analyzed various samples taken from the surfaces of a medical center for the treatment of COVID-19. The results showed the presence of viral RNA, indicating that the surfaces may be routes of transmission of infection [36]. An interesting study by Liu et al. showed that samples collected from hospital areas accessible only to medical staff showed higher amounts of viral RNA compared to samples collected from areas common to patients [37].

Currently, studies show that fomite transmission is circumstantial. For example, in a group of people with COVID-19 investigated associated with a mall in China, it was found that many people had no direct contact. However, it was observed that all persons used common facilities such as elevators, thus suggesting that the transmission took place through fomites [38]. Another important factor in direct contact transmission is hand hygiene. It has been found that proper hygiene as well as the use of disinfectants have been associated with decreased risk of infection. However, hand hygiene is correlated with better infection control in general, not particularly with SARS-CoV-2 [34].

*Other transmission routes*. Studies have shown the presence of SARS-CoV-2 in various biological fluids such as saliva or tears. To et al. evaluated a group of 12 people confirmed with COVID-19 and observed that in 11 of them the presence of the virus in saliva was detected by PCR, and in 3 patients, the virus was viable in saliva. These observations are important because SARS-CoV-2 could spread in other ways, such as kissing or other practices involving saliva exchange [39]. Regarding the presence of SARS-CoV-2 in tears, Xia and colleagues found its presence in 1 in 30 patients with COVID-19, but the virus could not be cultured in samples [40]. 

The fecal-oral transmission hypothesis emerged early in the pandemic and was related to the increased number of ACE2 receptors in the small intestine [41]. Zhang et al collected anal and oral samples from 16 patients with COVID-19. On day 0, they found detectable RNA in anal samples in four patients compared to eight patients where viral RNA was detected in oral samples. Although the viability of the virus has not been tested, it has been suggested that feces may be a source of infection [42].

Currently, there are no data supporting or refuting sexual transmission as a method of transmission. The analysis of vaginal fluid revealed the presence of viral RNA only in one study [43]. The RNA of viral strains was detected in 6 of 38 samples of semen by Li and co-workers. It is worth mentioning that three of the six patients died due to infection with SARS-CoV-2. Thus, it has been suggested that the presence of SARS-CoV-2 in semen may be a marker of disease severity [44]. On the other hand, vaginal and seminal fluids are unlikely to be a mode of transmission of SARS-CoV-2, and further studies are needed to assess this risk.

### 3.2. SARS-CoV-2 Penetration into the Cell

**Figure 3:**
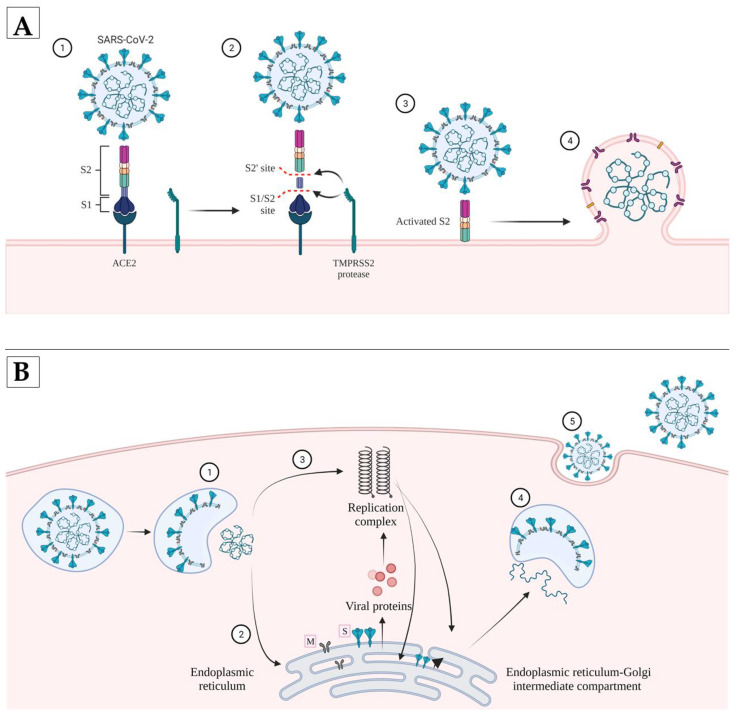
(**A**) Graphical representation of SARS-CoV-2 penetration inside the host cell. (1) Protein S has a transition to the metastatic-bile form, a lower energy state, before contact with the cell membrane; (2) Cleavage of the S protein at the S1/S2 site takes place with the separation of the receptor binding domain (RBD); (3) S2’ cleavage that causes fusion peptide exposure; and (4) Cell membrane-virus fusion and viral RNA release. (**B**) (1) Release of viral RNA inside the cell; (2) Part of the RNA is translated into viral proteins; (3) Viral proteins form a replication complex in order to form more RNA; (4) Proteins and RNA are assembled in the Golgi apparatus; and (5) Virion release. Created with BioRender.com (accessed on 29 March 2022).

In order to enter the host cell, the viral input proteins must fold into a stable energetic state, and then undergo a conformational transition to have enough energy to overcome the natural repulsion of the front cell membrane of the virus. Therefore, the S protein transitions from its stable state to its metastable state, which is a lower energy state, before it contacts the cell membrane. The S protein transition is activated by two-stage proteolytic cleavage. The first cleavage occurs at the S1-S2 boundary, and the second S2’ site in the S2 subunit. The pre-proteolytic cleavage at the S1-S2 position is performed by the furin in the virus-producing cell, while the cleavage of the S2’ site is performed by target cell proteases. The most important proteases involved in the activation of S protein are TMPRSS2 and cathepsin L (Figure 3). Thus, the penetration of the virus into the cell depends on the proteases of the target cell. The location of the two proteases is different from each other. TMPRSS2 is located on the cell surface, so the activation of the virus takes place in the cell membrane, while the activation mediated by cathepsin L takes place in the endolysosome [45]. Cleavage of S1-S2 activates proteins involved in the fusion of viral and cell membranes through several irreversible conformations, thus facilitating the penetration of the virus into the host cell. This is a complex process that involves proteolytic processing of the S protein and binding to the target receptor. Although there is a similarity between the SARS-CoV virus and SARS-CoV-2 in terms of the structure and preservation of the S protein, the presence of a furin cleavage site in the case of SARS-CoV-2 results in increased transmissibility to the virus [1].

According to structural studies, the main components of the fusion process of the cell membrane virus are the proteins FPPR, loop 630, and CTD2, which modulate fusogenic structural rearrangements of the protein S. FPPR and loop 630 are involved in maintaining the downward conformation of the RBD, and as the adjacent RBD faces upward, the FPPR and the 630 loop move into their positions [45]. If the ACE2 receptor binds the RBD-up conformation, the closed trimer S conformation is formed by removing the 630 loop and the FPPR. In this way the dissociation of S1 and the formation of the metastable form S2 take place, thus allowing the fusogenic transition to a stable postfusion structure. This cascade of events leads to the insertion of the fusion protein into the membrane of the target cell [46].

### 3.3. Target Receptors

*Angiotensin-converting enzyme 2 (ACE2)*. ACE2 is involved in the conversion of angiotensin to angiotensin II, playing an important role in the regulation of biological functions controlled by the renin-angiotensin system, such as the cardiovascular system. ACE2 plays a significant role in the process of virus penetration into the cell, as it is the primary receptor for S protein in SARS-CoV-2 [47].

Binding of the S protein to the ACE2 receptor occurs through the S1 subunit of the RBD. Compared to SARS-CoV, SARS-CoV-2 has an affinity up to 20 times higher for ACE2, which explained its increased infectivity [48]. The binding of protein S is performed at the level of type I of ACE2. In response to binding to the receptor, the virus forms a membrane fusion with the host cell. The virus then releases viral RNA into the cytoplasm [49]. SARS-CoV-2 competes with angiotensin II for ACE2 in receptor internalization. By binding the S protein to the receptor, it is blocked, the enzyme expression in the membrane is reduced, and the renin-angiotensin-aldosterone system is unbalanced [50]. Consequently, the decrease in angiotensin-mediated vasodilation coincides with the down-regulation of ACE2 and the onset of angiotensin-mediated vasoconstriction II [51].

A SARS-CoV-2 infection is associated with damage to a variety of organs since the ACE2 gene is expressed in almost every organ. ACE2 is predominantly found in alveolar cells type II, transient bronchial and respiratory epithelial cells, myocardial cells, smooth endothelial and muscle cells of the arteries, adipose tissue and others [48]. In addition, ACE2 expression has been linked to the potential risk of SARS-CoV-2 infection. Thus, tissues in which ACE2 is found in a proportion greater than 1% are correlated with a high risk. These tissues are represented by the respiratory tract, lungs, heart, ileum and others [52]. Moreover, ACE2 expression in the nasal epithelium varies with age, which may partially explain the higher prevalence of older people in SARS-CoV-2 infection [53].

*Other receptors.* Another SARS-CoV-2 receptor is cluster of differentiation 147 (CD147) [54], which is a transmembrane glycoprotein, widely expressed in tumor or inflamed tissues [55]. Multiple studies have shown the role of CD147 receptors. Thus, the study by Wang et al. found that the use of meplazumab, which is an inhibitor of CD147, blocks the blockade of cell invasion by SARS-CoV-2. In addition, in his study, Wang et. al. showed how the receptor binds to protein S [56]. Thus, the CD147 receptor is an alternative receptor for the penetration of SARS-CoV-2 into the cell and may also be a possible therapeutic target.

Neuropillin (NRP) is another potential receptor for SARS-CoV-2, facilitating the entry of the virus into the cell. NRP consists of two members, NRP1 and NRP2. NRP1 is a transmembrane protein that acts as a receptor for furin cleaved substrates [57]. It also acts as a regulator of the angiogenesis process under the action of endothelial vascular growth factor (VEGF) and participates in the control of cell migration in vessel remodeling [58]. NRP1 is expressed in both the respiratory epithelium and the olfactory epithelium, but also in endothelial cells, excitatory neurons, and epithelial cells in the nasal cavity. In COVID-19 patients, it was found that NRP1 showed increased expression and was suspected of facilitating viral invasion [59]. In the study by Daly et. al., it was observed that NRP1 binds protein S by the mechanism of the C-end canonical rule [60]. Regarding the penetration of the virus into the nervous system, it is assumed that this process is mediated by the interaction of the S protein and the receptor located in the olfactory system, the binding taking place in the b1/b2 domain of the receptor [61].

Dipeptidyl peptidase 4 (DPP4) is an ectopeptidase present in various cells of the immune system, kidney, lung or smooth muscle and contributes to various physiological and pathological processes mediated by the immune system [62]. The relationship between DPP4 and respiratory tract lesions was initially studied in MERS-CoV infection [63]. The study by Qi et al suggested that DPP4 acts as a receptor for SARS-CoV-2 with an expression pattern similar to the ACE2 receptor [64]. Murine studies of DPP4 receptor inhibition have shown a decrease in T cell count with a beneficial effect on the inflammatory process in the respiratory tract; however, further studies are needed on the role of DPP4 in COVID-19 patients [65].

## 4. Mutations in the SARS-CoV-2 Genome

### 4.1. Evolution of the Virus

It seems that SARS-CoV-2 was already present in nature long before the 2019 pandemic outbreak in China [66,67]. 

One of the hypotheses about the discovery of SARS-CoV-2 was issued by Sallard et al., according to which SARS-CoV-2 identified in a virus that appeared in 2012 in a mine in China, which was collected in a laboratory and which, due to an error, was dropped [68]. This hypothesis supports the theory that the virus existed in nature before the pandemic began. Another theory holds that SARS-CoV-2 is derived directly from the SARS-CoV virus, and the evolutionary analysis of SARS-CoV-2, SARS-CoV and MERS-CoV suggested that SARS-CoV-2 belongs to a new evolutionary branches of coronaviruses [69].

Regarding the evolution of the virus, studies have shown that the SARS-CoV-2 genome has many recombinant events. Although these recombination events have been identified throughout the genome, most of them have been detected in the ORF1a and N-terminus regions of protein S [70]. However, it has not yet been possible to identify ancestral genomes and highlighting the consequences of recombination events. From a pandemic perspective, protein S and RBD have been of great interest, since these proteins are critical for binding to ACE2 receptors and virus penetration into cells [71]. The evolutionary history of the S protein is complex, the sequence of this protein being composed of several segments that are phylogenetically related to the different strains of *Sarbecovirus* [70].

### 4.2. Mutations in the Spike (S) Protein and RBD

The COVID-19 pandemic has affected the entire human population since its outbreak in 2019. During the short time before the discovery of vaccines and effective monoclonal antibodies against SARS-CoV-2, there was hope that the disease could be controlled [72]. It is almost universal that monoclonal antibodies and vaccines target the S protein, and mutations at the level of the S protein have reduced the efficacy of these therapeutics [73]. For this reason, it is imperative to understand the mechanisms of viral mutations that occur at the level of the S protein, but also at other levels, for the development of therapeutic alternatives.

The process of mutation occurs as a result of competing processes that can occur at the molecular level, at the level of the organism or at the level of the population. At the molecular level, mutations lead to errors in the replication, transcription, translation, and transcription and translation processes, correction and viral recombination. Mutations in the body result from the adaptive immune response and recombination between the host and the virus, which produces changes in the host’s gene. Finally, mutations in the population are based on the principle of natural selection, which promotes viral reproduction and the spread of the virus [72].

The SARS-CoV-2 genome has nearly 29,000 mutations, but mutations in the S gene are of great importance because of the role that this protein plays in infectivity [74]. Specifically, there is a short immunogenic fragment in the S protein called the receptor binding domain (RBD) that facilitates the binding of the S protein to ACE2 [75]. 

Protein S is involved in the penetration of SARS-CoV-2 into the host cell, thus playing a crucial role in infectivity and pathogenicity [76]. The S protein consists of two subdomains, S1 and S2. S1 consists of an N-terminal domain (NTD) and a C-terminal domain (CTD). These domains function as RBDs, contributing to the recognition and binding of ACE2 receptors. At the same time, S2 contributes to the fusion between the virus and the host cell through conformational changes. S1 has wide variations, while the S2 domain is the most stable region of the S protein [77].

There are six major subtypes of SARS-CoV-2 characterized by the recurrent D614G mutation of the spike protein that have evolved globally as a result of the continuous evolution of the virus. Mutational variants of the S protein cause virus variants that are resistant to the immune responses of CD4 + T cells or resistant to long-term immune protection involving T cells memory [78]. The D614G mutation involves the substitution of residue 614 for aspartic acid with glycine. An increase in viral RNA content was detected in the upper respiratory tract in patients with the D614G mutation, as well as an increase in infectious capacity [79].

After the D614G mutation, another mutation, N439K, appeared globally. This mutation involves a substitution of amino acids in the binding factor to the receptor, being of real importance due to the increase in the binding affinity of the ACE2 receptor and the decrease in the neutralizing activity of monoclonal and polyclonal antibodies [14]. The RBM amino acid mutation Y453F has also gained attention due to its high affinity for ACE2. In addition, this mutation has been associated with COVID-19 cases identified in both humans and mink [80].

**Figure 4:**
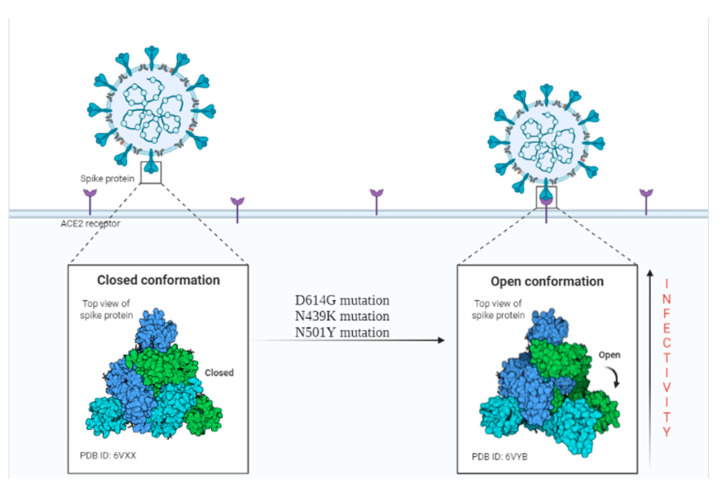
Presentation of the closed and open conformation of the S protein, the mutations appeared at this level determining the adoption of the open position, necessary for the binding to the ACE2 receptors, which leads to the increase of the infectivity. Created with BioRender.com (accessed on 29 March 2022).

At the end of 2020, another mutation took place in the genome of SARS-CoV-2, N501Y. This mutation has led to three “501Y” lines of care, including V1 (Alpha), V2 (Beta) and V3 (Gamma), all of which are of global concern due to their high transmission rate [81]. SARS-CoV-2 N501Y.V1 has 17 mutations, most of which are localized at the S protein level, while SARS-CoV-2 N501Y.V2 has 10 mutations at the S protein level and 3 mutations at the RBD level. Both types have a higher rate of transmissibility and better resistance to neutralizing antibodies compared to the original strain [82]. Therefore, understanding the mutations in S protein and RBD promotes understanding of the infectivity, transmission, and evolution of SARS-CoV-2, contributing to the success of vaccines and treatments. Figure 4 shows the two conformations (closed and open) of the S protein that may be influenced by mutations at this level.

### 4.3. N-terminal Domain (NTD) Mutations

The N-terminal domain of Spike protein exhibits recognized epitopes of neutralizing antibodies produced by the host’s immune system. In addition, this region of the S protein is involved in the adhesion process at the cell surface [83].

NTD deletions observed over time in the SARS-CoV-2 genome have been described as changes in antigenicity [84]. Four recurrently erased regions (RDRs) of the NTD were identified, and five separate deletions were frequently studied at their level, namely RDR, RDR1, RDR2, RDR3 and RDR4. Deletions RDR2 and RDR4 blocked the binding of the neutralizing monoclonal antibody 4A8 [85]. An additional proof of the role played by RDR2 deletions in avoiding the immune response is the appearance of Δ140 in SARS-CoV-2 which causes a marked decrease in the neutralization titer. Furthermore, there was a fourfold reduction in non-neutralizing titers as a result of the consecutive appearance of the E484K mutation at the RBD level. Thus, by associating mutations in RBD and NTD, variants can be obtained that can drastically avoid the response of the polyclonal antibody [86].

### 4.4. Mutations Related to the Host Immune System

More than half of the C > T mutations in the genome of SARS-CoV-2 are due to the host immune system, which is mediated through the catalytic apolipoprotein mRNA editing enzyme system (APOBEC). The ratio of C > T mutations increases with age, thus explaining the ratio of infections among different categories of people [87].

The study by Jaroszewski et al. found that there was an excess of mutations in the NSP1 and NSP2 proteins and also in the ORF (3a, 8b and 14), all these proteins being involved in the interactions between the virus and the host [88]. It has also been shown that a mutation in the NSP1 protein can increase the vulnerability of the virus to immune clearance [89]. At the same time, NSP3 is involved in the inflammatory process associated with COVID-19 by the release of cytokines. As a consequence of NSP3 expression in interferon-activated macrophages, proinflammatory genes are expressed for a prolonged period, causing cytokine storms to occur, which are characteristic of severe forms of COVID-19 [90].

According to the study by Wang et al., the NSP6 L37F mutation, initially found in Asia, has been associated with an increase in the number of asymptomatic cases [91]. NSP6 alters the process of atophagy of infected cells, causing cell death [92]. Moreover, between January and May 2020, it was observed that the rate of mutations in protein S, NSP12 and NSP13 increased until April, after which they decreased. On the other hand, some parts of the genome showed an increase in the mutation rate even after April [93]. These mutations occur in N protein, especially R203K and G204R mutations, and in viroporin 3a, ORF3a, G251V and Q57H mutations. These viral proteins can bypass the interferon-induced immune response because protein N, protein 3a and NSP6 are antagonists of interferon β [92].

### 4.5. SARS-CoV-2 Variant

**Figure 5:**
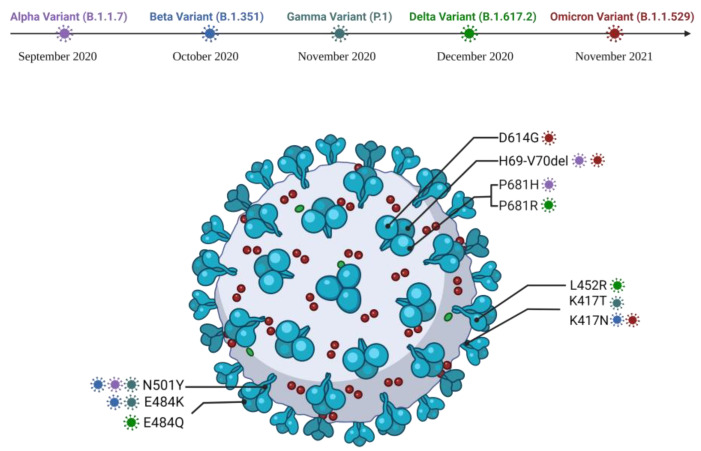
Variants of concern for SARS-CoV-2 occurred from December 2020 to the present. Created with BioRender.com (accessed on 30 March 2022).

A characteristic feature of viruses is mutation, in which they acquire superior properties like greater infectiousness and transmissibility, until they finally become dominant. Figure 5 shows the five variants of concern. The SARS-CoV-2 virus is no different from other types of viruses, which is why, since the beginning of the pandemic, its genome has undergone several mutations with an impact on infectivity, immune response and treatment [94].

#### 4.5.1. Alpha Variant

The Alpha variant (B.1.1.7) was first identified in the United Kingdom in September 2020, and later, it spread widely, globally. From the genome point of view, this variant shows Δ69/70 and Δ144 in the NTD and the N501Y mutation at the RBD level [95]. In terms of resistance to highly neutralizing antibodies, this variant has a minimum sensitivity. However, the fusogenic potential is higher for this variant, thus leading to increased mortality and transmissibility [96,97]. Compared to D614G, the Alpha version predicts an improved host cell fusion. In vitro studies have shown that 501Y mutation improves in vitro binding affinity through hydrophobic interactions between RBD Y501 and ACE Y41 and favorable cation-π stacking interactions with ACE2 K353. In addition, compared to D614G, existing vaccines are effective, providing a long-term protective effect [98].

#### 4.5.2. Beta Variant

The Beta variant (B.1.351) was first identified in South Africa in May 2020 and has since become widespread. This variant is characterized by the presence of K417N, E484K and N501Y mutations in the spike protein and amino acid deletions of position 241–243 [98]. These deletions, together with N501Y and E484K mutations, are associated with a lower binding of neutralizing monoclonal antibodies and human serum antibodies [99]. It has been shown that the E484k mutation favors the electrostatic interactions of RBD with ACE2, with studies suggesting that SARS-CoV-2 variants with this type of mutation have a significantly higher affinity for ACE2 receptors, which contributes to a higher rate of transmission [100]. The current vaccines have a lower efficacy on this variant compared to the Alpha and D614G variants [101]. 

#### 4.5.3. Gamma Variant

The Gamma variant (P.1) was first identified in Brazil in November 2020 and is a descendant of the Beta variant. This variant has mutations similar to the Beta mutation in RBD, such as N501Y, K417T/N and E484K. In contrast, the Gamma variant is less resistant to naturally acquired or vaccine-induced antibodies compared to the Beta variant [102]. Both Beta and Gamma show changes in the C-terminal end of the S protein, but the implications of these mutations are unknown. As for the existing vaccines, they have a corresponding efficacy against this variant [98].

#### 4.5.4. Delta Variant

The Delta variant (B.1.617.2) was first identified in India in October 2020 and is part of the progeny of SARS-CoV-2 B.1.617 together with the Kappa variants (B.1.617.1) and B.1.617.3, all these variants presenting various mutations at the level of the Spike protein, RBD and NTD [98]. Initially identified in India in 2020, the Delta strain has spread widely ever since. This variant has 11 mutations specific to the Spike protein, of which the P618R mutation is highly conserved, which facilitates the cleavage of furin and gives the virus a high fusogenicity and pathogenicity [103]. Although the Alpha variant has a missense mutation at position 681 amino acids with a histidine residue (P681H), the Delta variant is much more transmissible than the Alpha variant [104]. Most of the mutations in the Delta variant are localized to the binding surface of RBD to ACE2, thus avoiding neutralization by antibodies [105]. In addition, the Delta variant has mutations in the NTD, which results in the remodeling of the antigenic surface of the NTD loop and a change in the glycane shield around the antigenic site. These changes result in total resistance to NTD-specific neutralizing antibodies [101]. All vaccines, both mRNA-based and adenoviral-based vaccines, generate an appropriate immune response only after the second dose [98].

#### 4.5.5. Omicron Variant 

The Omicron variant (B.1.1.529) was first identified in Botswana in November 2021. This variant has 15 mutations at the RBD level, 7 mutations at the NTD level and 3 mutations in the vicinity of the furin cleavage site [98]. Studies have shown that mutations in Omicron’s S protein cause the virus to become more transmissible than the Delta variant. Thus, the N501Y mutation increases the binding affinity of RBD to ACE2 and, in addition, the combination of the N501Y and Q498R mutation increases this affinity, leading to an increase in infectivity. Regarding the efficacy of monoclonal antibodies, such as Tixagevimab and Regdanvimab, they have lost their antiviral activity against this variant, while ADG-20 and Sotrovimab are active [106]. As far as the effectiveness of vaccines is concerned, they are ineffective after two doses; however, when administered three times, the effectiveness reaches over 70% [107]. 

## 5. Impact of the SARS-CoV-2 Mutation

### 5.1. Impact on Diagnostics

One of the important steps in managing the COVID-19 pandemic is to detect the virus quickly and accurately. Diagnostic methods include reverse transcription polymerase chain reaction (rRT-PCR), which enables detection of SARS-CoV-2 nucleic acids in naso-pharyngeal fluid [108]. Testing has been developed to prevent the spread of a virus to the community. Misdiagnosis and negative results may cause public health problems [109]. In this regard, the improvement of test sensitivity and specificity remains a priority. On the other hand, serological tests detect possible previous infections, having an important role from a therapeutic point of view. Antibodies are detected by enzyme-linked immunosorbent assay by quantitative detection of IgG and IgM antibodies [110]. These tests can be used to determine the immune response against the viral protein S and to assess the protection against further viral exposure [111]. 

**Figure 6:**
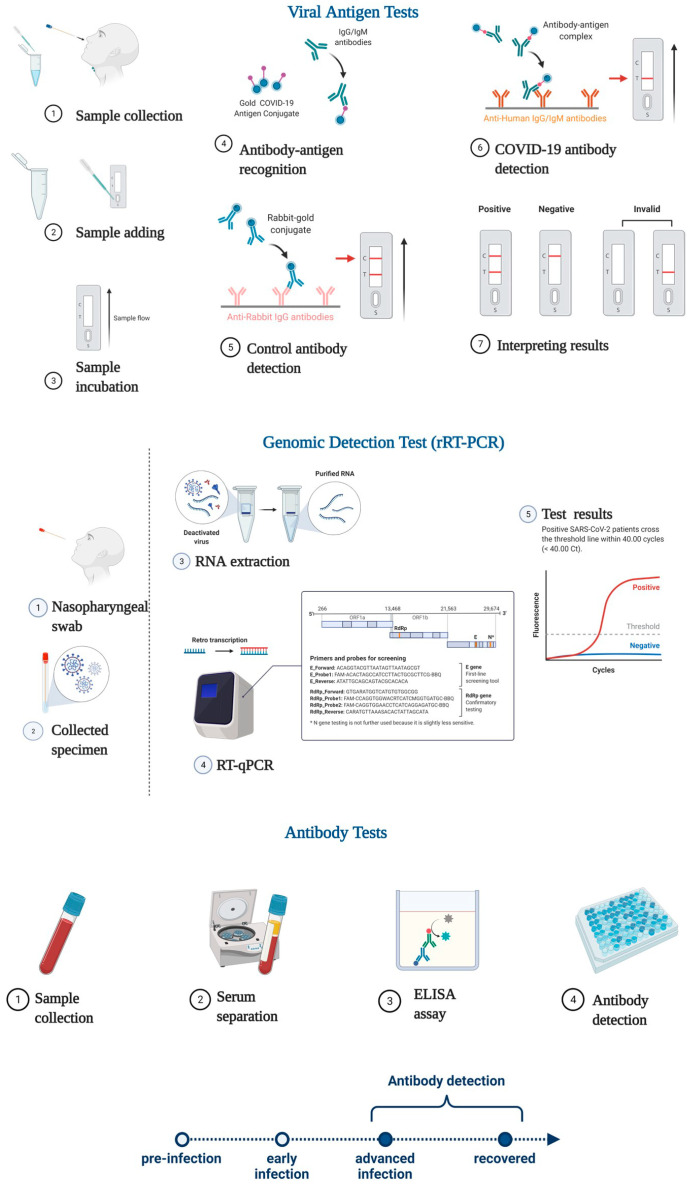
Methods used to diagnose COVID-19. Created with BioRender.com (accessed on 30 March 2022).

Currently, the detection of SARS-CoV-2 and the diagnosis of COVID-19 is based on three types of tests: nucleic acid tests, viral antigen tests and antibody tests (Figure 6). 

Genomic testing for SARS-CoV-2 includes two main technology platforms, namely, real-time reverse transcription polymerase chain reaction (rRT-PCR) and high-throughput sequencing. In addition to these methods, other PCR tests are also used, such as loop-mediated isothermal amplification (LAMP-PCR). Genomic testing has improved sensitivity and specificity for viral detection compared to serological testing. Selective identification of SARS-CoV-2 against other respiratory viruses is mainly by RT-PCR which proves that this method is sensitive, accurate and specific. The procedure is based on the isolation and conversion of viral RNA into complementary DNA (cDNA). Following this, the cDNA is amplified using Taq DNA polymerase, and then the viral load is quantitatively measured [112].

RT-PCR is considered the gold standard for SARS-CoV-2 detection, which involves identifying several genes in the virus such as protein S, N, E, NSP12 or NSP14 [113]. The qPCR method used for detecting SARS-CoV-2 virus is a technique where mainly the N and S genes of the virus are identified. Given that these genes have a high probability of mutation, the accuracy of the tests may be affected [114]. Regarding the spike protein, it has unique nucleotide sequences for SARS-CoV-2 minimizing cross-reactivity and false-positive results in the presence of other types of Coronaviruses. The S protein of SARS-CoV-2 bears approximately 76% similarity to that of SARS-CoV in amino acid sequence. In addition, compared to other Spike CoV proteins, the RBD region of SARS-CoV-2 has almost 25% more mutations. Further, the uniqueness of the S protein of SARS-CoV-2 is manifested in the S1 domain which has an identity of approximately 50% with SARS-CoV and in the S2 domain which has an identity of approximately 90% with SARS-CoV. Moreover, differences between the S proteins of SARS-CoV-2 and SARS-CoV appear in the 3D structure, the SARS-CoV-2 protein being composed of a larger number of helices and sheets, and the binding to hACE2 receptors is more rigid in the case of SARS-CoV-2 [115]. It is important to validate diagnostic tests regularly to avoid false negative results, since many mutations can occur in the S protein. An incorrect diagnosis was described for Alpha and Omicron variants, despite using protein S, protein N, and ORF1ab as well. Although the N protein is not as prone to mutations, any type of mutation that occurs in the primer binding region can interfere with the sensitivity and accuracy of the assay [116]. A study by Vanaerschot et al. reported that the N protein Q289H mutation in the N protein affects the binding of the primer and significantly reduces the sensitivity of the RT-PCR assay [117]. 

One of the alternatives to the RT-PCR test is the isothermal amplification, which is based on the thermal cycle [118]. Simplified RT-PCR is used to detect several regions of the SARS-CoV-2 genome such as protein S, RNA polymerase (RdRp)/helicase (Hel) proteins or N proteins [108]. As for RdRp/Hel tests, they have the advantage of increased sensitivity for viral identification, providing fast and reliable results [42]. The RT loop-mediated isothermal amplification (RT-LAMP) method is based on nanotechnology. LAMP diagnostic tests are performed either by turbidity levels or by colorimetric or fluorescence measurements. This technique is easy to perform and generally has little background interference [112].

Other types of tests have also been developed, such as a rapid diagnostic test to detect the presence of SARS-CoV-2 viral antigens in the respiratory tract. This test is based on binding the antigen in the sample to antibodies attached to a paper tape. This reaction generates a visible signal only if the virus reproduces actively. Therefore, this type of test can be used to diagnose acute or early infection [112]. In terms of antigen tests, they can be performed in public places for the purpose of screening the population or they can be used by patients independently. It is mainly based on the detection of viral antigen epitopes (N or N + S protein) by enzyme-linked immunosorbent assays (ELISAs) [119]. If the virus is rapidly evolving, tests targeting a single epitope have a lower sensitivity and, consequently, a lower accuracy of the results [120]. Thus, in the case of the Omicron variant, it has been observed that N mutations can determine the accuracy of currently approved commercial antigen tests [121]. 

The primary immune response to SARS-CoV-2 infection is manifested by antibody synthesis. Serological studies can be both an alternative to RT-PCR tests and a complement to them for significantly increasing viral detection rates. IgM antibody levels increase in the first week of SARS-CoV-2 infection, reaching a maximum of two weeks post-infection, after which they return to a relatively normal level. On the other hand, IgG antibodies are detectable one week after infection and are maintained at a high level for a long time, serving as protection against reinfection [122]. Antibody tests are used to detect serum antibodies produced by the immune response to SARS-CoV-2 or a vaccination. The S and N proteins have the highest immunogenicity, which is why most tests detect S or N-directed antibodies [123,124]. Mutations at this level may impact the test because the patient’s antibodies may no longer recognize the new mutant structure.

### 5.2. Impact on Therapeutics

**Figure 7:**
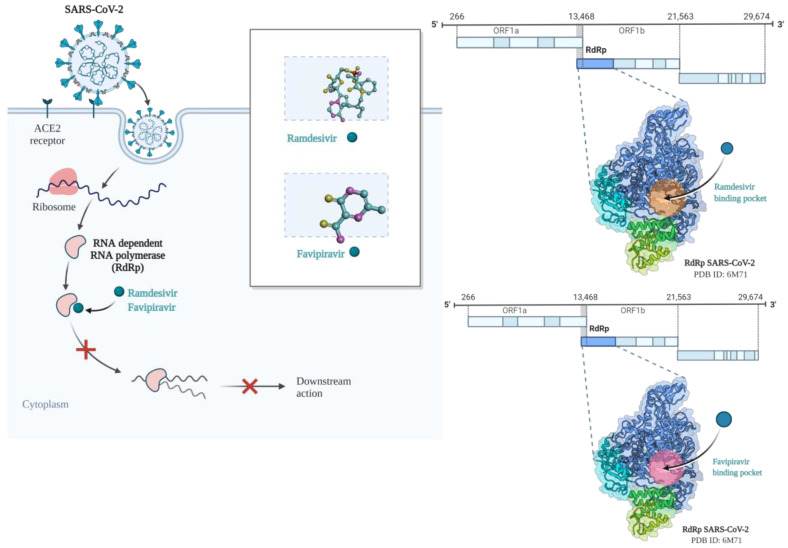
Schematic representation of the mechanism of action of Ramdesivir and Favipiravir antivirals. Created with BioRender.com (accessed on 30 March 2022).

COVID-19′s first-line treatment includes broad-spectrum antivirals such as Remdesivir and Favipiravir (Figure 7) and anti-inflammatory drugs to reduce the cytokine storm in severely ill patients [116]. 

Various small molecules can be used in targeted therapy that target viral structures involved in virus penetration into the cell or cellular replication, such as Spike protein, RNA-dependent RNA polymerases (RdRp) or nucleocapsid and nsp5 [125]. The main small molecules used in SARS-CoV-2 therapy are the therapeutic target of RdRp and include antiviral drugs such as Remdesivir and Favipiravir [126,127]. Silico studies have shown that the occurrence of various mutations in RdRp may interfere with Remdesivir binding [125]. In addition, alanine at position 156 in RdPb has been shown to play a crucial role in binding to the drug, so any mutation at this level may interfere with the effectiveness of the antiviral [128].

Therapeutic antibodies have the advantage of high specificity due to the mechanism of action similar to that of natural antibodies [129]. The combination of Casirivimab-Imdevimab monoclonal antibodies presents as a therapeutic target two epitopes of the S protein; thus, Casirivimab targets the spike-like loop from the top direction (overlapping with the ACE2-binding site) and Imdevimab binds the S protein RBD from the front or lower-left side, being active on most circulating variants [129,130]. However, in vitro tests on Casirivimab have shown that, when used alone, it is less effective on mutants such as Delta and Gamma. As far as the Omicron variant is concerned, Casirivimab is partially active while Imdevimab is inactive, and the combination of the two does not appear to be effective [131]. 

The combination of the monoclonal antibodies Bamlanivimab and Etesevimab also presents as a therapeutic target two different epitopes of protein S. In the RBD of the S protein of SARS-CoV-2, Bamlanivimab and Etesevimab bind to distinct, but overlapping, epitopes. While Bamlanivimab binds to a RBD epitope in both its open and closed conformations, Etesevimab binds to the up/active conformation of the RBD [129]. Although this combination is effective on Alpha and Delta variants, on Beta and Gamma variants, the neutralization activity is diminished [116].

Tocilizumab is a recombinant humanized monoclonal antibody in which initial studies have shown improvement in hospitalized COVID-19 patients [132]. Subsequent reports have shown that tocilizumab may reduce mortality in severely COVID-19 patients [133]. However, the role of tocilizumab in hospitalized patients who are not in critical condition remains unclear.

In the case of a rapidly changing pandemic virus, it is necessary to identify the viral strain before administering the monoclonal antibody, a rather difficult task. For this reason, the administration of a single antibody is not necessarily an appropriate therapeutic strategy, as mutations in the genome can quickly become resistant to that antibody. For this reason, the use of polyclonal antibodies or monoclonal antibody combinations is a more appropriate therapeutic strategy [116]. 

In addition to these therapies, several methods have been introduced to modulate the disordered immune response in patients with severe COVID-19. These methods include the use of interferons, corticosteroids and anti-inflammatory cytokines [134].

Interferon is a key inflammatory cytokine in SARS-CoV-2 infections [135]. In general, interferons are used in certain types of cancers and hepatitis C [136,137]. Research has shown that the use of IFN-α/β in patients with SARS or MERS does not bring benefits [138]. However, in combination with SARS-CoV-2 infection, the combination of IFNβ-1b and lopinavir/ritonavir is more effective than using lopinavir/ritonavir alone in reducing symptoms and shortening hospital stays [139]. To date, there are insufficient studies on the toxicological profile of interferons, which is why their use is recommended only in severe cases of COVID-19.

One of the goals of anti-COVID-19 therapy is to reduce the plasma levels of pro-inflammatory cytokines by using corticosteroids [140,141]. Dexamethasone is one of the compounds of the corticosteroid class recommended for SARS-CoV-2 infection. In spite of the fact that studies suggest dexamethasone can significantly reduce mortality, it is not without side effects, such as an impairing effect on the ability to eliminate the virus and an increased risk of secondary infection [142,143,144]. Another corticosteroid used is methylprednisolone. It has the advantage of a quick start action and a half-life of 24–36 h. Additionally, studies have demonstrated that the use of methylprednisolone has reduced mortality in patients over the age of 60 [145].

Another therapeutic approach for SARS-CoV-2 is chloroquine and hydroxychloroquine. As for hydroxychloroquine, it works by increasing the endosomal pH, thus inhibiting the fusion between SARS-CoV-2 and the host cell membranes [146]. On the other hand, chloroquine inhibits the glycosylation of the ACE2 receptor, thus interfering with the binding of the virus to the cell membrane [147]. Although in vitro studies have shown an immunomodulatory effect of the two substances, clinical trials have shown that hydrochloride does not reduce mortality, but may increase the length of hospitalization [148,149]. In addition, no benefit was observed with the combination of hydroquinone and azithromycin [150]. Thus, neither chloroquine nor hydroxychloroquine were recommended by the COVID-19 Treatment Guideline Panel [134].

Ivermectin is an antiparasitic drug approved by the World Health Organization and the US Food and Drug Administration, used predominantly in low- and middle-income countries. With the onset of the SARS-CoV-2 pandemic, observational and randomized trials evaluated the efficacy of ivermectin in both treatment and prophylaxis. An analysis by the Front Line COVID-19 Critical Care Alliance concluded that ivermectin has strong therapeutic efficacy against SARS-CoV-2. A recent analysis also showed that ivermectin significantly reduced COVID-19 deaths. However, the National Institutes of Health in the United States has stated that there is insufficient scientific evidence for the use of the drug, and the World Health Organization recommends against its use outside of clinical trials [151].

Mainly, neither antivirals nor anti-inflammatory drugs are affected by mutations, but mutations have adverse effects on targeted therapy [116].

### 5.3. Impact on Vaccines

To date, COVID-19 is prevented by five vaccine types, namely: (i) Live-attenuated or inactivated vaccine; (ii) use of the protein subunit; (iii) viral vector; (iv) vaccine containing mRNA and plasmid DNA; and (v) vaccine with virus-like particles [152]. Given that SARS-CoV-2 is an RNA virus, it has a much higher mutation rate than DNA viruses, which affects the effectiveness and reactivity of neutralizing antibodies [153].

There are currently two mRNA vaccines available, Pfizer and Moderna. The advantage of these vaccines in mutations in the SARS-CoV-2 genome is their easy ability to target only SPIKE protein and relatively easy fabrication [154]. Although the effectiveness of the two vaccines against infection has decreased with the onset of mutations, studies show that they are still effective in preventing severe symptoms and hospitalization [155]. Because of the many mutations that can occur in Omicron, vaccine effectiveness has been questioned. Experiments on the Pfizer vaccine have shown a substantial decrease in the potency of neutralizing this variant [156].

Adenoviral vector vaccines have the Spike protein as their target and have the major disadvantage of the possibility of the vaccinated individual developing immunity against adenovirus. Currently approved adenoviral vector vaccines are Oxford-AstraZeneca, CanSinoBio (Convidecia), Janssen and Sputnik V (Gam-COVID-Vac) [157]. All of these vaccines, with the exception of CanSinoBio, where there are no reports of efficacy to date, have shown a decrease in the ability to neutralize Beta variant [158,159,160]. As for the Omicron variant, Oxford-AstraZeneca and Janssen vaccines show a decrease in neutralizing capacity [159].

Although the disadvantages of inactivated virus vaccines are their increased production time and low immunogenicity, the many advantages they offer against SARS-CoV-2 make them an important strategy for immunizing the population. Thus, inactivated vaccines elicit a polyclonal antibody response to several viral antigens, such as the S and N proteins [161].

## 6. Conclusions and Future Directions

Viral evolution is a continuous phenomenon, resulting in the selective adaptation of viral forms. In addition, RNA viruses have as their predominant feature the high mutation rate that causes changes in the transmission process, virulence, host immune response and receptor binding affinity. Currently, there is evidence of mutations in spike protein and amino acids that affect antibody neutralization. A full understanding of the impact and consequences of mutations in the SARS-CoV-2 genome will help elucidate the transmission process and provide solutions for the prevention and treatment of COVID-19.

One of the main reasons for the rapid spread of SARS-CoV-2 is the appearance of asymptomatic forms and mild forms that are not properly diagnosed. In light of this, it is becoming increasingly difficult to follow people who are infected; therefore, it is inevitable that mutations will occur. Although the development of vaccines has been a rapid process since the beginning of the pandemic, the emergence of mutations that can prevent the immune response has led to pandemic waves. Therefore, further efforts are needed to continuously identify the mutations that have occurred and to increase the capacity for testing and sequencing. 

The study and understanding of the SARS-CoV-2 genome was comprehensively studied, facts that participated in the molecular diagnosis and development of vaccines and targeted therapies. However, there are concerns about variations in the SARS-CoV-2 genome that could affect targeted therapies and the development of vaccines. For this reason, additional molecular research into the SARS-CoV-2 genome is a global priority for the development of new, more sensitive genetic detection methods, the implementation of new broad-spectrum antiviral drugs, and the development of new vaccines based on the genetic profile of both the virus and the host cell. General awareness and active implementation of prevention measures at the individual level is also crucial. Both medical staff and the general public need to be regularly updated on mutations and their impact on diagnosis, therapy and vaccines to ensure proper medical management, on the well-known principle that it is better to prevent to treat. 

## Data Availability

Not applicable.
